# *Megacraspedus cottiensis* sp. nov. (Lepidoptera, Gelechiidae) from northern Italy – a case of taxonomic confusion

**DOI:** 10.3897/zookeys.963.54842

**Published:** 2020-08-24

**Authors:** Peter Huemer, Ole Karsholt, Christian Wieser

**Affiliations:** 1 Tiroler Landesmuseen Betriebsges.m.b.H., Sammlungs- und Forschungszentrum, Naturwissenschaftliche Sammlungen, Krajnc-Str. 1, A-6060 Hall, Austria Tiroler Landesmuseen Betriebsges.m.b.H. Innsbruck Austria; 2 Zoological Museum, Natural History Museum of Denmark, Universitetsparken 15, DK-2100 Copenhagen, Denmark Natural History Museum of Denmark Copenhagen Denmark; 3 Landesmuseum für Kärnten, Sammlungs- und Wissenschaftszentrum, Liberogasse 6, A-9020 Klagenfurt, Austria Landesmuseum für Kärnten Klagenfurt am Wörthersee Austria

**Keywords:** brachyptery, Cottian Alps, DNA barcoding, morphology, misidentification, new species

## Abstract

*Megacraspedus cottiensis***sp. nov.** is described from the western Alps (prov. Torino, Italy). The dorsal habitus and genitalia for both the male and brachypterous female are provided. The new species belongs to the *M. faunierensis* species group based on genitalia morphology and DNA barcodes, and was hitherto confused with *M. neli* Huemer & Karsholt, 2018 from the southwestern Alps. However, it clearly differs in morphology and DNA barcode sequences from that species and from *M. faunierensis* Huemer & Karsholt, 2018. The new species is suspected of being a regional endemic of the Cottian Alps.

## Introduction

*Megacraspedus* comprises 85 described species, therefore one of the most diverse genera of Palearctic Gelechiidae. It was recently revised by Huemer and Karsholt (2018) with 44 species introduced as new to science. Almost half of the genus (46 species) are diagnosed from only male specimens and the biology of the majority is unknown. Species diversity in the genus, not associated or incorrectly associated males and females, and limited biological knowledge has created some recent problems in identification and classification. Fortunately, descriptions of the unknown females for five species were recently provided (Nel and Varenne 2019), and additionally, likely overlooked or unknown species were detected (Corley pers. comm.) as a result of this work.

As part of a survey of the fauna of Lepidoptera from the Cottian Alps (northwest Italy), a large number of specimens identified as *M. neli* were collected, including associated female specimens which were unknown when the species was described. *Megacraspedus neli* Huemer & Karsholt, 2018 was described based on the male holotype collected in the south of France and two male paratypes from the Cottian Alps (northwestern Italy). It therefore seemed reasonable to publish the newly discovered, brachypterous female in a short note. However, examination of the genitalia of both sexes revealed that the specimens identified as *M. neli* collected in the new survey from the Cottian Alps differed morphologically from the holotype. The relevant diagnostic characters were overlooked as a result of the *M. neli* description (Huemer and Karsholt 2018) being based on the genitalia of a paratype in glycerol. These characters are more easily recognised in permanent preparations. The new species hypothesis was corroborated by comparing the DNA barcode of the holotype of *M. neli*, obtained using Next Generation Sequencing methods, to the DNA barcode sequences from specimens collected in the Cottian Alps. The new species is therefore described below.

## Materials and methods

The study is based on 248 specimens of the *Megacraspedus faunierensis* species group: *M. faunierensis* (127 specimens), *M. neli* (1 specimen), and *M. cottiensis* sp. nov. (120 specimens). Specimens were pinned, wings spread, and dried. Genitalia preparations followed standard techniques (Robinson 1976) adapted for male genitalia of Gelechiidae by the “unrolling technique” as described by Pitkin (1986). In the absence of properly set specimens, the forewing length measured from the base to the apex of fringes is given instead of the wingspan (Huemer and Karsholt 2018).

Habitus photographs were taken with an Olympus SZX 10 binocular microscope and an Olympus E-3 digital camera. Images were z-stacked using the software Helicon Focus 4.3 and digitally edited in Adobe Photoshop CS4 and Lightroom 2.3. Genitalia photographs were taken with an Olympus E-1 Digital Camera on an Olympus BH2 microscope.

DNA samples were extracted from dried legs in order to sequence the 658 base-pair long barcode segment of the mitochondrial COI gene (cytochrome c oxidase 1) according to the prescribed standards. In addition to specimens previously treated by Huemer and Karsholt (2018), legs from five specimens of the *M. faunierensis* species group were successfully processed at the Canadian Centre for DNA Barcoding (CCDB, Biodiversity Institute of Ontario, University of Guelph) to obtain DNA barcodes using the standard high-throughput protocol described in deWaard et al. (2008). The DNA sequence of the holotype of *M. neli* was recovered using Next Generation Sequencing techniques (Prosser et al. 2016). New sequences were submitted to GenBank, and further details including complete voucher data and images can be accessed in the public dataset “DS-MEGAFAUN *Megacraspedus faunierensis* species group” https://dx.doi.org/10.5883/DS-MEGAFAUN in the Barcode of Life Data Systems (BOLD systems v. 4.0. http://www.boldsystems.org; Ratnasingham and Hebert 2007, Ratnasingham 2018). Degrees of intra- and interspecific variation of DNA barcode fragment were calculated under Kimura 2-parameter model of nucleotide substitution using analytical tools of BOLD. Neighbour-Joining analysis from previously published sequences of *Megacraspedus* (Huemer & Karsholt, 2018) and the additional samples was conducted in MEGA7 (Kumar et al. 2016).

Abbreviations of specimen repositories:

**LMK** Landesmuseum Kärnten, Klagenfurt, Austria

**TLMF** Landesmuseum Kärnten, Klagenfurt, Austria

**ZMUC** Zoological Museum, University of Copenhagen, Denmark

## Taxonomy

### *Megacraspedus faunierensis* species group

The *M. faunierensis* species group includes three species: *M. neli*, *M. faunierensis*, and *M. cottiensis* sp. nov. Male genitalia are characterised by the proportionally small uncus as compared to the tegumen, the straight and long gnathos hook, the longitudinal medial ridge of the saccular area, the oblong saccus with a longitudinal medial ridge, and the shape of the phallus with wrinkles. Females of *M. faunierensis* and *M. cottiensis* are brachypterous but unknown for *M. neli*. For detailed morphological descriptions and diagnoses, see Huemer and Karsholt (2018).

#### Megacraspedus cottiensis sp. nov.

Taxon classificationAnimaliaLepidopteraGelechiidae

0F9D2C15-3475-56F9-9A52-ED24CEDABF04

http://zoobank.org/16E7C5ED-3271-473B-B5D7-B48D50128A20

Figures 2–3 , 4 , 7

##### Type material.

***Holotype.*** Italy; • ♂; prov. Torino, Parco Naturale N Orsiera – Rocciavrè, Fenestrelle, ca. 0.7 km NE Pequerel; 45°3'6"N, 7°4'16"E; 1820 m; 29 Jun. 2019; leg. P. Huemer; [Barcode identification number] TLMF Lep 27447; [genitalia slide number] GEL 1299 ♂ P. Huemer; coll. TLMF (Fig. 2).

##### Paratypes.

Italy; • 39 ♂, 2 ♀; same data as holotype; 1 ♂; [Barcode identification number] TLMF Lep 27448; 1 ♀; [Barcode identification number] TLMF Lep 27446; [genitalia slide number] GEL 1300 ♀ P. Huemer; coll. TLMF; • 31 ♂, 2 ♀; same data as holotype; 23 Jul. 2019; coll. TLMF; • 10 ♂; same data as holotype; leg. C. Wieser; coll. LMK; • 7 ♂; prov. Torino, Parco Naturale N Orsiera – Rocciavrè, Via Colle delle Finestre, Forte Serre Mariae E; 45°2'58.88"N, 7°3'5.29"E; 1840 m; 30 Jun. 2019; leg. C. Wieser; coll. LMK; • 1 ♂; prov. Torino, Valsusa, Mompantero, Monte Rocciamelone; 2200 m; 3 Jul. 1993; leg. G. Bassi; genitalia prep. (in glycerin); coll. ZMUC [misidentified paratype of *M. neli*]; • 1 ♂; same data, but loc. Riposa; 2200 m; 16 Jul. 1993, leg. G. B. Delmastro; coll. TLMF [misidentified paratype of *M. neli*]; • 5 ♂; prov. Torino, Parco Naturale Orsiera – Rocciavrè, Usseaux, Colle delle Finestre N, 45°4'21"N, 7°3'11"E; 2180 m; 24 Jul. 2019; leg. P. Huemer; coll. TLMF; • 14 ♂; prov. Torino, Parco Naturale Gran Bosco di Salbertrand, 2 km SE Colle dell´Assieta; 45°3'38"N, 6°58'44"E; 2240 m; 25 Jul. 2019; leg. P. Huemer; coll. TLMF; • 5 ♂; prov. Torino, Parco Naturale Gran Bosco di Salbertrand, 1.8 km SE Colle dell´Assieta; 45°3'40"N, 6°58'21"E; 2350 m; 21 Jul. 2020; leg. P. Huemer; coll. TLMF.

##### Diagnosis.

*Megacraspedus cottiensis* belongs to a group of species with three distinct black spots in the forewing. It is characterised by the dark basal part of the costa and the dark grey-brown costal area of the forewing in the males, but otherwise it is very similar to other species of the *M. faunierensis* species group. This species is also similar to *M. tristictus* Walsingham, 1910 and *M. pentheres* Walsingham, 1920 in external appearance.

Major diagnostic characters are found in the male genitalia (Figs 4–6). The male genitalia of *M. cottiensis* differs from that of *M. neli* by the slightly narrower uncus, the long lateral sclerites of the saccus exceeding the maximum width of the saccus, and the slender phallus. The new species is easily separated from *M. faunierensis* by the smaller, less triangular-shaped saccus with proportionally longer lateral sclerites, and several other subtle characters such as the proportionally larger uncus, and more slender valva and phallus.

The female genitalia differ from that of *M. faunierensis* by the convex anterior projection of the subgenital plate and the transverse, suboval signum whereas characters such as the differing lengths and widths of the ductus and corpus bursae may be variable (Figs 7, 8). The female genitalia are similar to those of several other species of *Megacraspedus* and the documented characters generally seem of limited diagnostic value in the delimitation of species.

**Figure 1.**
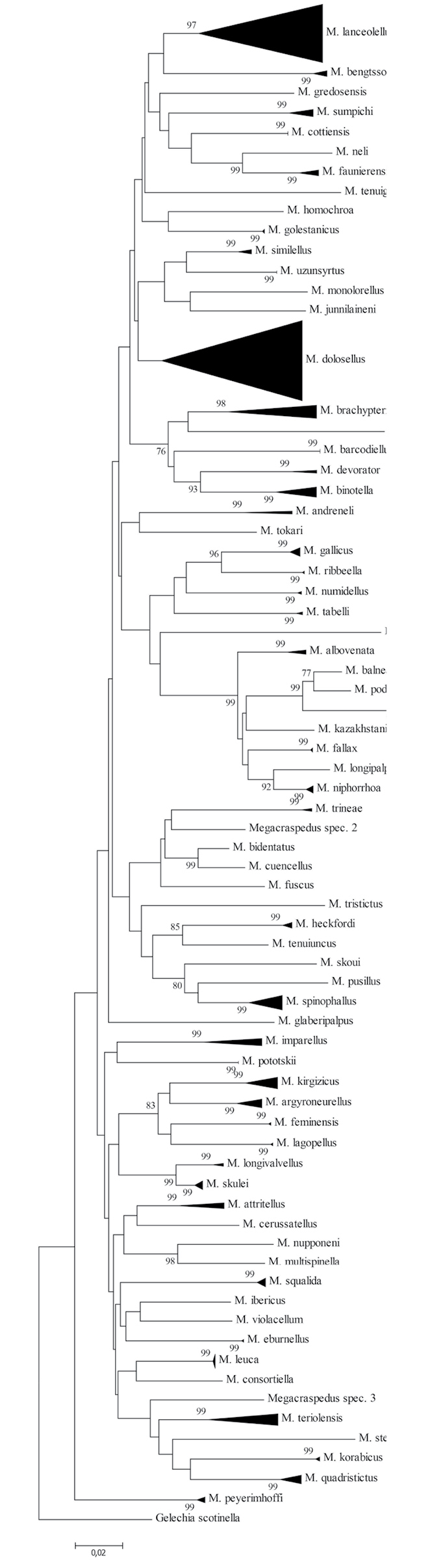
Neighbor-Joining tree (built with MEGA7) of cytochrome c oxidase subunit I (COI) barcode fragments. Values on branches represent bootstrap values of ≥ 75 % inferred from 500 replicates, scale bar represents substitutions per site. Note: the scale bar only applies to internal branches between species. The width of the triangles represents the sample size, and the depth the relative genetic variation within the cluster (2× scale bar). Source: DNA Barcode data from BOLD (Barcode of Life Database, cf. Ratnasingham and Hebert 2007).

Finally, all species are easily separated by DNA barcode sequences (Fig. 1).

##### Description.

**Figures 2–3.**
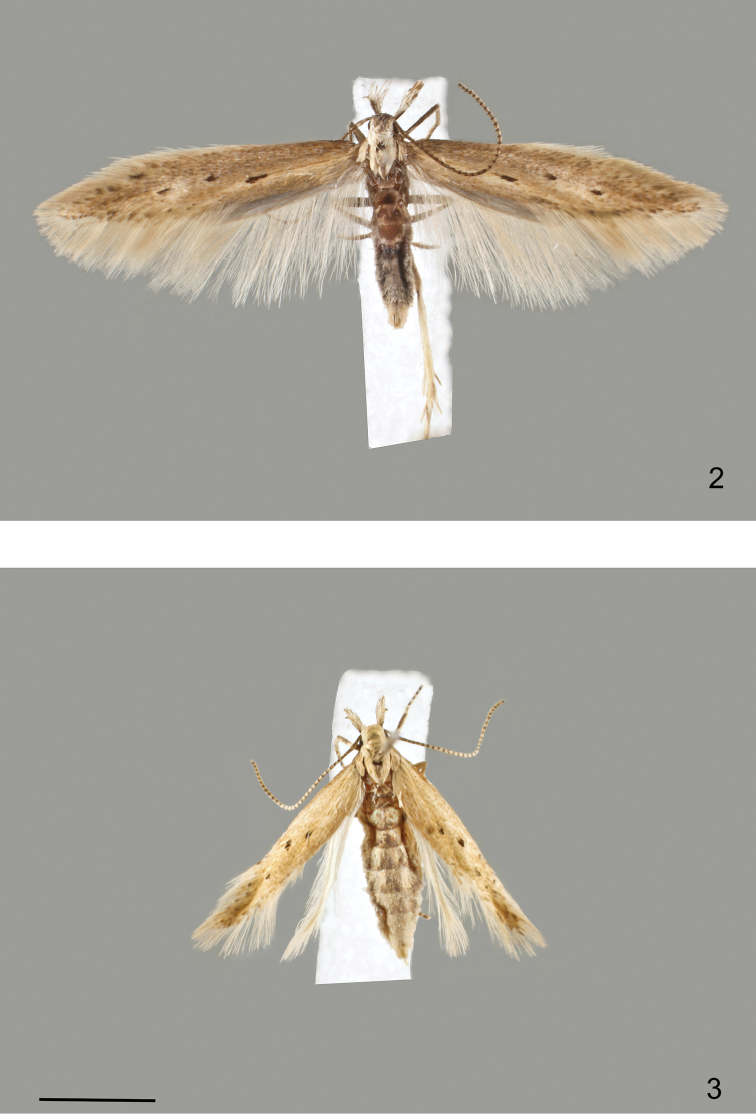
*Megacraspedus cottiensis* sp. nov., adult. **2** male, holotype; **3** female, paratype. Scale bar: 2 mm, all to scale.

Adult. Male (Fig. 2). Forewing length 4.0–5.8 mm. Segment 2 of labial palpus with moderately long scale brush, brown on outer surface, white mottled with brown on inner surface, white on lower and upper surface; segment 3 creamy white. Antennal scape without pecten; flagellum dark brown, at most weakly ringed with white. Head and thorax creamy white to cream. Forewing light yellowish brown, more or less intensively mottled with brown, costal half variably covered with grey-brown scales, dorsal half lighter, base of costa dark grey-brown, a black dot in fold at 2/5 and two black dots in middle of wing and at end of cell; some black-tipped scales along termen; fringes light grey. Hindwing light grey with concolourous fringes. Female (Fig. 3). Flagellum distinctly ringed with white. Head and thorax yellowish brown. Forewing length 4.1–4.2 mm. Forewing narrow, apically pointed, almost unicolourous light yellowish brown, without grey brown costal half, darker towards apex, fringes whitish. Hindwing lanceolate, whitish grey. Otherwise similar to male.

Variation. The extension of the darkened part of the costa as well as the amount of black scales on the forewings is variable.

**Figures 4–6.**
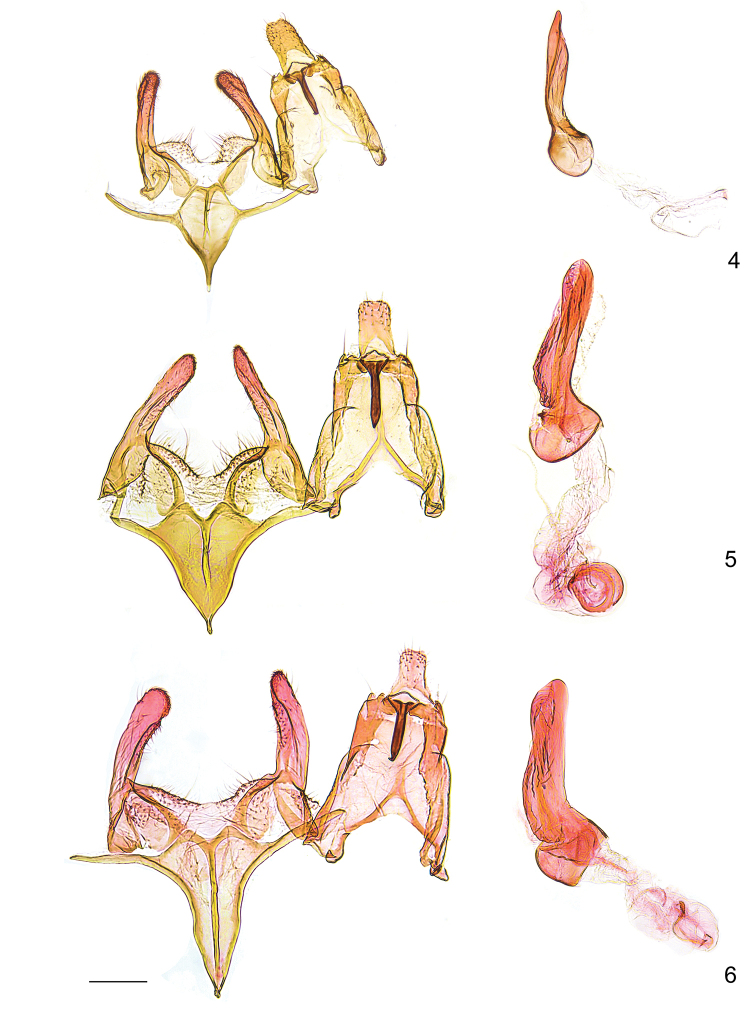
Male genitalia of *Megacraspedus faunierensis* species group. **4***M. cottiensis* sp. nov., holotype; **5.***M. neli*, holotype; **6***M. faunierensis*, paratype, genitalia slide GEL 1219. Scale bar: 200 µm, all to scale.

*Male genitalia* (Fig. 4). Uncus moderately small, nearly sub-rectangular, apical corners rounded, apical edge with weak medial emargination; gnathos hook moderately slender, straight, apically pointed, approximately length of uncus; anterior margin of dorsal surface of tegumen with suboval emargination, sclerotised ridges from anterior edge converge in medial part of tegumen; pedunculi small, suboval, with small ridge; valva approximately width of uncus, stout, extending slightly beyond base of uncus, digitate distal part, apex broadly rounded; saccular area densely covered with setae, with longitudinal ridge, without separated sacculus; posterior margin of vinculum medially emarginated, with lateral humps, vincular sclerite elongated sub-ovate, with nearly straight sclerotised posterior edge; saccus moderately small, slightly shorter than valva, slender V-shaped, ratio maximum width to length 0.6, posterior margin with weak convex projections, separated by minute incision, medial part with sclerotised ridge from posterior margin to approximately middle, lateral sclerites approximately 1.3 times length of maximum width of saccus; phallus weakly curved at ca. one-third, with inflated coecum, two times wider than distal part, distal part 2.5 times length of coecum, sclerotised dorsal ridge, apex slender; ductus ejaculatorius with small internal sclerotisation.

**Figures 7–8.**
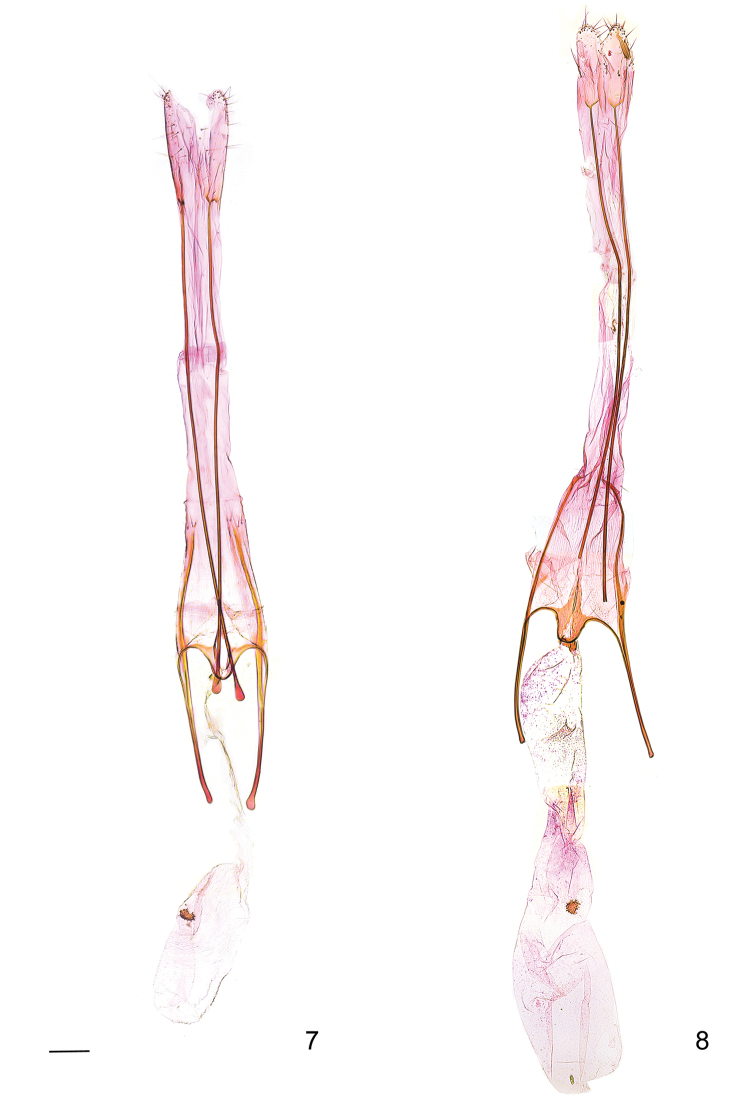
Female genitalia of *Megacraspedus faunierensis* species group. **7***M. cottiensis* sp. nov., paratype, genitalia slide GEL 1300; **8***M. faunierensis*, paratype, genitalia slide GEL 1235. Scale bar: 200 µm, all to scale.

*Female genitalia* (Fig. 7). Papilla analis small, apically rounded; apophysis posterior slender rod-like, ca. 2.5 mm long, with short, bifurcate posterior end, bordered by small sclerotised field; segment VIII long and slender, ca. 0.7 × 0.4 mm, largely membranous; subgenital plate with sub-triangular subostial sclerotisation, posteriorly weakly extended sclerites delimiting small ostium bursae, anterior margin with rod-like edge connected with apophysis anterior, medially with moderately short convex projection; apophysis anterior slender, rod-like, free-standing part approximately length of segment VIII, posteriorly becoming rod-like venula of segment VIII, extending to posterior margin of segment VIII; colliculum short; ductus bursae short, slender; corpus bursae, moderately short and slender, distinctly delimited from ductus bursae, entire length of ductus and corpus bursae ca. 1.7 mm; signum small, transverse, suboval spiny plate.

##### Distribution.

Northern part of the Cottian Alps in northwestern Italy.

##### Biology.

**Figure 9.**
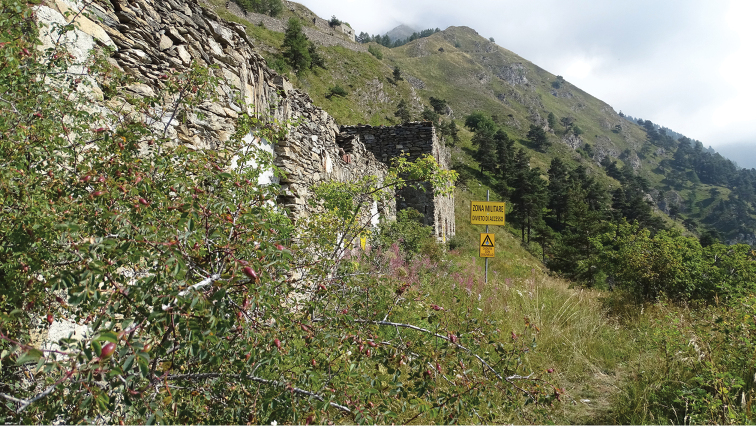
Habitat of *Megacraspedus cottiensis* sp. nov., Parco Naturale Orsiera – Rocciavrè, Forte Serre Mariae environs (photograph M. Vilgut).

Host plant and early stages are unknown. Adults were collected from late June to late July in a xeromontane grassland (Fig. 9) at artificial light sources. Males were attracted in the first 3 hrs of the night, with females, discovered by lighting the vegetation with a headlamp. Specimens were collected at altitudes ranging from ca. 1800 to 2350 m.

##### Etymology.

The specific name is an adjective derived from the Cottian Alps, where the new species was discovered.

##### Molecular analysis.

Eight specimens of the *Megacraspedus faunierensis* species group, covering all three described species, were successfully sequenced (sequence length 599 to 658 bp). Intraspecific distances are generally low (not applicable for *M. neli*), ranging from a minimum of 0% to a maximum of 1.71%, whereas interspecific divergence is much higher, ranging between 7.12 and 9.04% among members of the species group and 12.14% to the nearest species *M. sumpichi* Huemer & Karsholt, 2018 (Table 1, Fig. 1) These values, however, are based on a low number of samples. All species in the group belong to different BINs (Ratnasingham and Hebert 2013); the BIN ID for *M. cottiensis* is BOLD:AEA4020 (n = 3). All the individuals of the new species share the same haplotype and the distance to the nearest congeneric neighbour, *M. faunierensis*, is 9.04% (p-dist).

**Table 1.** Intraspecific mean K2P (Kimura 2-parameter) divergences, maximum pairwise distances and distance to Nearest Neighbour in the *Megacraspedus faunierensis* species group.

Species	Mean Div.	Max Div.	Nearest Species	Nearest Neighbour	Distance to NN
*Megacraspedus faunierensis*	1.01	1.71	*Megacraspedus neli*	DEPAL068-20	7.12
*Megacraspedus neli*	N/A	N/A	*Megacraspedus faunierensis*	LEASU040-18	7.12
*Megacraspedus cottiensis*	N/A	N/A	*Megacraspedus faunierensis*	LEASU040-18	9.04
*Megacraspedus sumpichi*	N/A	N/A	*Megacraspedus cottiensis*	LEASV695-19	12.14

## Discussion

We were surprised to discover another undescribed species of *Megacraspedus* in the Alps given the recent revisionary treatment (Huemer and Karsholt 2018), but, at the same time, it supports the hypothesis of even more species diversity and local endemism in a region where the genus is already rich. *Megacraspedus cottiensis* is another apparently small-scale endemic to the western Alps. The difference between this species’ DNA barcode and its nearest neighbour, *M. faunierensis*, is quite high for these taxa (ca. 9%); and this despite their geographic proximity to each other (i.e., the type localities are only ca. 70 km apart.). There is a high probability that the divergence from a possible common ancestor pre-dates the last glacial period. It is precisely for taxa such as these that the need for standardised morphological examinations and the greatest possible completeness of DNA barcode reference libraries are evident. By way of example, in this study, the successful morphological differentiation of the new species from closely related taxa was corroborated with the DNA barcode of the holotype of *M. neli* made possible only by NGS methods. Other equally rich and enigmatic taxa may benefit from similar sampling and methodological approaches.

## Supplementary Material

XML Treatment for Megacraspedus cottiensis
